# An RNAi screen for ribosome biogenesis genes required for *Drosophila* border cell collective migration

**DOI:** 10.17912/micropub.biology.001292

**Published:** 2024-08-10

**Authors:** Emily Burghardt, Jocelyn A. McDonald

**Affiliations:** 1 Division of Biology, Kansas State University, Manhattan, Kansas, United States

## Abstract

Ribosome biogenesis is critical for the proper production of proteins in cells and has emerged as a regulator of cell invasion and migration in development and in cancer. The *Drosophila* border cells form a collective that invades and migrates through the surrounding tissue during oogenesis. We previously found that a significant number of ribosome biogenesis genes are differentially expressed from early to late migration stages. Here, we performed a small-scale RNAi screen of a subset of these ribosome genes. Knockdown of seven genes disrupted border cell migration, thus revealing a role for ribosome biogenesis genes in regulating collective cell migration.

**Figure 1. Differentially expressed ribosome biogenesis genes are required for border cell migration:**
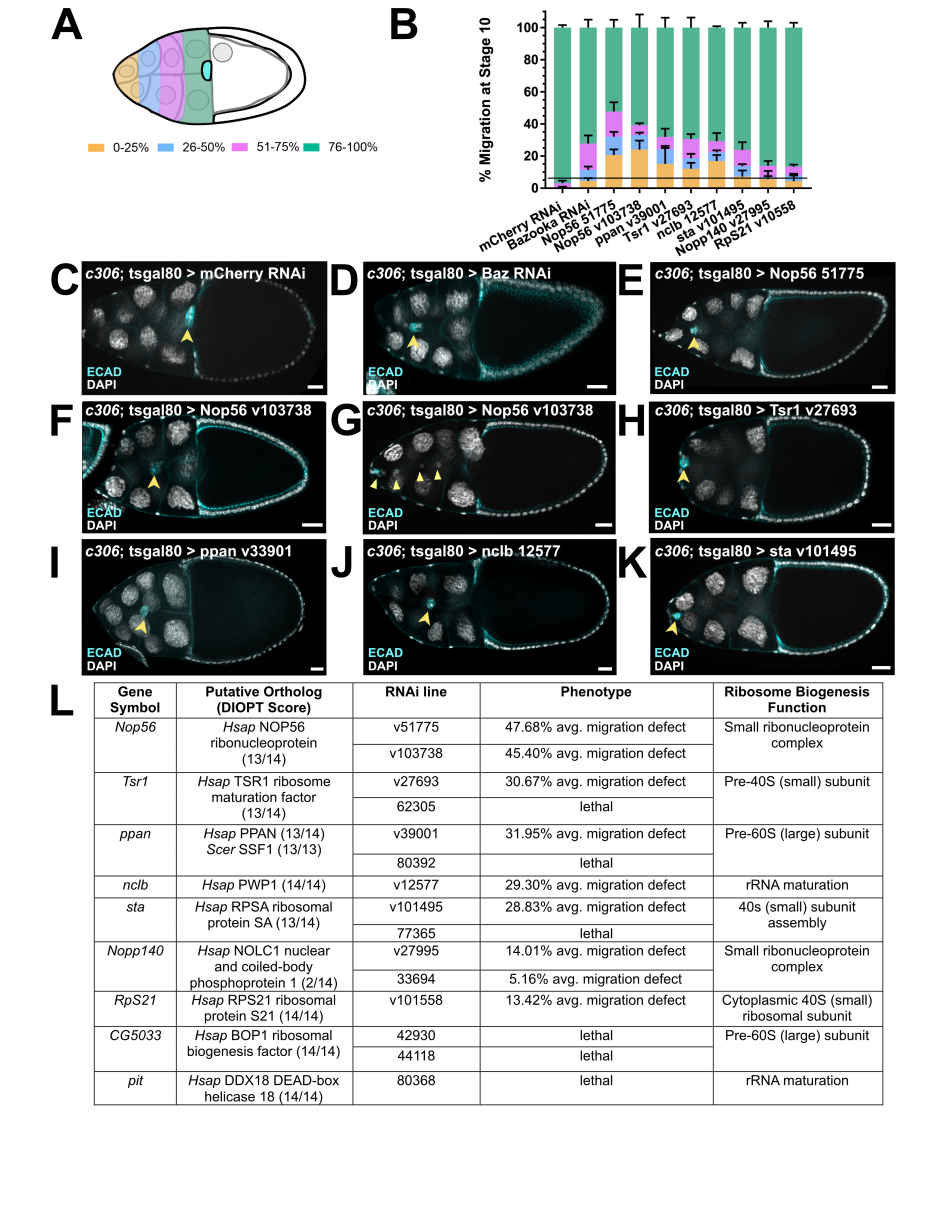
(A) Schematic illustrates scoring of migration defect in border cells upon RNAi knockdown of ribosome biogenesis genes. Migration defect is scored as a percentage of the total migration distance to the oocyte (0-25%, orange; 26-50%, blue; 51-75%, pink; 76-100%, green). (B) Quantification of border cell migration defects upon knockdown of ribosome biogenesis genes compared to knockdown of *mCherry* RNAi (negative control, left). The migration defect upon *mCherry* RNAi knockdown was used to determine the threshold for significance of migration defects, represented as a black line (6% migration defect; see Methods). Error bars represent standard deviation. (C) RNAi knockdown of mCherry in border cells results in complete migration by stage 10 of oogenesis. (D) RNAi knockdown of *Bazooka* (positive control) in border cells results in migration defects at stage 10 of oogenesis. (E-K) RNAi knockdown of significantly differentially expressed genes with roles in ribosome biogenesis results in migration defects: *Nop56* (E-G); *Tsr1* (H); *ppn* (I); *nclb* (J); or *sta* (K). Cluster splitting was observed for RNAi knockdown of *Nop56* with one line (G). E-cadherin (E-cad) labels the border cell cluster (in cyan) and DAPI labels cell nuclei (in white). Yellow arrowheads denote the border cell cluster (C-F, H-K) or individual border cells (G). All scale bars represent 20 μm. (L) Table details the genes chosen with ortholog information (see Methods), RNAi lines used, phenotype (average [avg.] migration defect or lethality), and gene functions in ribosome biogenesis. Two genes had no phenotypes when knocked down by RNAi: *RpLP0* and *RpL17* (see Reagents Table).

## Description

During collective cell migration, small to large groups of cells move together as a cohort instead of as individual cells. Collective cell migration is essential for the proper development of tissues and organs and helps close wounds (Friedl & Gilmour, 2009; Scarpa & Mayor, 2016) . Abnormal collective cell migration helps drive tumor metastasis and disrupts wound healing (Cheung & Ewald, 2016; Friedl & Gilmour, 2009) . Characterization of the molecular mechanisms that underlie collective cell migration is therefore essential for our understanding of both normal development and diseases such as cancer. Recent increases in access to transcriptomics have allowed researchers to analyze the changes in gene expression that shape collective cell migration (Bae et al., 2017; Kasemeier-Kulesa et al., 2023; Morrison et al., 2017; Olson et al., 2024) . These transcriptome studies provide both a global view of transcriptional changes in cells during migration and a wealth of gene candidates for additional functional analyses. *Drosophila* border cells are a genetically tractable model of collective cell migration within an intact tissue (Montell et al., 2012) . In a bulk RNA sequencing study, we identified differential expression of transcriptional networks that were up- or down-regulated in the small cluster of border cells during their collective migration through the developing egg chamber, the major subunit of the ovary (Burghardt et al., 2023) . Our transcriptome analysis highlighted networks of genes expressed in border cells during migration, including genes involved in mechanisms known to regulate collective cell migration, such as the actin cytoskeleton and cell-cell adhesion (Burghardt et al., 2023) . Additionally, we found an unexpected enrichment of differentially expressed genes with annotated roles in ribosomes (87 genes; Burghardt et al., 2023). Further bioinformatic analyses confirmed that the majority of these genes (52 genes) have predicted or functional roles in ribosome biogenesis, with an additional 30 genes involved in ribosome translation or RNA modifications in the cytoplasm (Burghardt et al., 2023) .

To investigate the function of ribosome biogenesis and related genes in border cell migration, we chose representative genes from each of the major differentially expressed functional categories: ribosomal RNA (rRNA) maturation, ribonucleoprotein (RNP) complex formation, and ribosomal large and small subunit formation (Burghardt et al., 2023; Ni & Buszczak, 2023a). Genes chosen for functional testing additionally had a demonstrated role in the ribosome in *Drosophila* or had a high confidence ortholog (Alliance of Genome Resources Consortium, 2024; Öztürk-Çolak et al., 2024), and had readily available RNAi reagents (Reagents Table; Figure 1 ). Using these criteria, we chose 11 ribosome biogenesis genes to test for function in border cell migration. We drove RNAi knockdown using the *c306-* GAL4 driver, which is expressed in follicle cells and border cells prior to and during migration (Aranjuez et al., 2016) . We assessed border cell migration at stage 10 of oogenesis, by which time border cells should have reached the oocyte boundary and stopped their migration ( Figure 1A ). Migration was scored as a percentage of the total distance migrated to the oocyte (0-25%, 26-50%, 51-75%, or 76-100%; Figure 1A, B ). RNAi knockdown of *mCherry* , which encodes a fluorescent protein not normally found in *Drosophila* , served as a negative control; *mCherry-* RNAi did not impair movement, resulting in complete border cell migration at stage 10 of oogenesis ( Figure 1B, C ). An RNAi line against *Bazooka* , a gene known to be required for border cell migration, was used as a positive control (Pinheiro & Montell, 2004) ; *Baz-* RNAi disrupted migration in 28% of egg chambers ( Figure 1B, D ).

Hundreds of proteins are required for the assembly of the small of large ribosomal subunits that comprise a mature, functional ribosome (Ni & Buszczak, 2023a). This process begins with the association of assembly factors and proteins with ribosomal RNA (rRNA), forming ribonucleoprotein (RNP) complexes. Of the genes tested with roles in RNP complex formation, knockdown of two ( *Nop56* and *Nopp140* ) resulted in significant migration defects. Knockdown of Nop56 function in border cells with either of two RNAi lines resulted in strong migration defects ( Figure 1B, E -G). In some cases, Nop56 knockdown resulted in a specific cluster splitting defects, in which border cells split from the main cluster while migrating forward ( Figure 1G ). The RNAi line in which this defect was observed (v103738) has a predicted potential off-target effect to *Hormone receptor 4* ( *Hr4* ); Hr4 is required in border cells for their migration (Manning et al., 2017) . However, we observed strong migration defects upon *Nop56* knockdown with two non-overlapping RNAi lines, including a line that is not predicted to impact *Hr4* . Further work, including live imaging, will be needed to assess the nature of the cluster splitting defect caused by *Nop56* knockdown in border cells. Knockdown of a second gene in RNP complex assembly, *Nopp140* , resulted in a significant migration defect with one of two RNAi lines ( Figure 1B, L ). In *Drosophila* larval cells, loss of *Nopp140* results in abnormal RNP granule formation (He et al., 2015) , though this remains to be tested in border cells.

Ribonucleoprotein (RNP) complex formation is essential for ribosomal subunit formation. Each large (60S) and small (40S) ribosomal subunit is itself a complex of ribonucleoproteins and ribosomal RNAs (Dörner et al., 2023; Ni & Buszczak, 2023a). Knockdown of three genes with functional roles in ribosomal subunit assembly (Ameismeier et al., 2018; Marygold et al., 2007; Zielke et al., 2022) , *Tsr1 ribosome assembly factor* ( *Tsr1* ), *peter pan* ( *ppan* ), and *stubarista* ( *sta* ), resulted in significant border cell migration defects with one RNAi line ( Fig. 1B, H, I, K, L ). For each gene, knockdown using a second RNAi line resulted in lethality, likely due to earlier functions during *Drosophila* development ( Figure 1L ). After initial transcription in the nucleolus, pre-ribosomal RNA must be folded, processed, and modified. As the precursors to RNP formation and subunit assembly, proper rRNA maturation represents an essential first step (Baßler & Hurt, 2019) . Knockdown of one gene with a role in maturation of ribosomal RNA (rRNA), *no child left behind* ( *nclb* ), also resulted in a significant migration defect ( Figure 1J, L ). Knockdown using one RNAi line for a second gene with a role in rRNA maturation, *pitchoune* ( *pit* ), was lethal ( Figure 1L ). Finally, RNAi for two other ribosomal genes, *RpLp0* and *RpL17* , did not impair border cell migration, though only one line per gene was tested (see Reagent Table). Ribosome biogenesis is a critical process in all cells, especially during rapid growth and differentiation (Ni & Buszczak, 2023a, 2023b). Therefore, the use of additional GAL4 drivers and non-overlapping RNAi lines will be needed to confirm the migration defects and lethality described here.

Here, we report a functional role for a subset of ribosome biogenesis genes that are differentially expressed in border cells during their migration. Knockdown of seven genes resulted in significant migration defects. Recently, the ribosome has emerged as a potential regulator of invasive and migratory cell behaviors (Costa et al., 2023; Prakash et al., 2019; Sherwood et al., 2023) . Changes in ribosome biogenesis, including expression of ribosomal proteins, are associated with increased mRNA translation. Prior to invasion of the anchor cell during *C. elegans* vulval development, an increase in ribosome biogenesis provides an increase in translation to establish high levels of required cytoskeletal, adhesion, and signaling proteins in the cell (Costa et al., 2023; Sherwood et al., 2023) . Similarly, upregulation of ribosome biogenesis is associated with the epithelial-to-mesenchymal transition and invasive potential of tumor cells (Prakash et al., 2019) . Our data represent new candidate differentially expressed genes required in border cells during their migration, with which to understand the role of ribosome biogenesis in migratory cell collectives during development and disease.

## Methods

*Drosophila RNAi screen and genetics*

All fly stocks and crosses were maintained at 25°C. Genes were chosen to test from the list of significantly differentially expressed ribosome biogenesis genes in border cells (Burghardt et al., 2023) . The ortholog information displayed in Figure 1L was identified using the Drosophila RNAi Screening Center Integrative Ortholog Prediction Tool (DIOPT) high-confidence putative ortholog data from FlyBase; “ *Hsap* ” and “ *Scer* ” represent *Homo sapiens* and *Saccharomyces cerevisiae* , respectively (Hu et al., 2021, 2011; Öztürk-Çolak et al., 2024). For RNAi screening, stocks were obtained from the Bloomington Drosophila Stock Center (BDSC) and the Vienna Drosophila Resource Center (VDRC). Where possible, two independent, non-overlapping RNAi lines were chosen per gene. All RNAi lines tested are listed in the Reagents Table. Virgin females from a *c306-* GAL4; ts-gal80/CyO stock were crossed to UAS-RNAi males to drive expression of the knockdown construct in border cells. RNAi against *mCherry* (BDSC 35785) was used as a negative control, and *Bazooka* RNAi ( *Baz* ; VDRC v2914) was used as a positive control. Female progeny from these crosses were selected and fattened on wet yeast paste overnight at 29 °C to allow maximal GAL4/UAS expression and inactivation of ts-GAL80.

*Immunostaining and imaging*

To analyze border cell migration, whole ovaries were dissected in Schneider’s *Drosophila* medium (Thermo Fisher Scientific) and fixed in 4% methanol-free formaldehyde (Polysciences) and 0.1 M potassium phosphate buffer (pH 7.4) for 10 minutes. Egg chambers were stained for E-cadherin (E-cad; 1:10 dilution, rat monoclonal DCAD2; Developmental Studies Hybridoma Bank [DSHB]), Singed (Sn; 1:25 dilution, mouse monoclonal Sn7C; DSHB), and DAPI to label nuclei (2.5 μg/mL; Millipore Sigma, cat. no. D9542). Isotype-specific anti- mouse or anti-rat secondary antibodies conjugated to AlexaFluor–488 or –568 (Thermo Fisher Scientific) were used at a concentration of 1:400 (see Reagents Table). All samples were mounted on slides using FluorSave Reagent mounting media (Millipore Sigma, cat. no. 345789). Migration defects were analyzed, and images were obtained, on an upright Zeiss AxioImager Z1 with ApoTome2 using a 20X objective and an Axiocam 503 mono camera. All images were processed in FIJI and assembled in Affinity Designer (RRID:SCR_016952).

*Graph and quantification*

Three trials per line were used with at least 25 egg chambers scored per trial (27 ≤ *n* ≤ 193 egg chambers). Singed and E-cadherin were used to evaluate migration distance ( Figure 1A ), but only E-cadherin is shown in Figure 1 . The minimal cutoff value for evaluating an overall migration defect was determined by the “background mean migration defect,” which was calculated using an average of the migration defect that was observed in the negative control ( *c306* -Gal4; tsgal80/+; UAS- *mCherry* RNAi/+). To assess the significance of migration defects, the empirical rule for a normal distribution was used, where the threshold for having a significant migration defect was equal to the mean plus three times the standard deviation. The graph in Figure 1B was created using GraphPad (version 7.04; RRID:SCR_000306). The graphing schematic and figure were created and assembled in Affinity Designer (RRID:SCR_016952).

## Reagents

**Table:** 

**Organisms/strains**				
***Drosophila* Strains **	**Genotype**	**Identifier**	**Source**	
*c306-* GAL4; ts-GAL80	P{w[+mW.hs]=GawB}c306, w[1118]; w[*]; P{w[+mC]=tubP-GAL80[ts]}20	RRID:BDSC_3743 and RRID:BDSC_7019	Made by McDonald lab from BDSC stocks	
UAS- *mCherry* RNAi	y[1] sc[*] v[1] sev[21]; P{y[+t7.7] v[+t1.8]=VALIUM20-mCherry.RNAi}attP2	RRID:BDSC_35785	Bloomington Drosophila Stock Center (BDSC)	
UAS- *Baz* RNAi	w[1118]; P{GD1384}v2914	FBst0457833	Vienna Drosophila Resource Center (VDRC)	
UAS- *Nop56* RNAi	w[1118]; P{GD8135}v51775	FBst0469571	VDRC	
UAS- *Nop56* RNAi	P{KK102280}VIE-260B (v103738)	FBst0475596	VDRC	
UAS- *ppan* RNAi	w[1118] P{GD11766}v39001	FBst0462794	VDRC	
UAS- *ppan* RNAi	y[1] sc[*] v[1] sev[21]; P{y[+t7.7] v[+t1.8]=TRiP.HMC06628}attP40	RRID:BDSC_80392	BDSC	
UAS- *Tsr1* RNAi	w[1118] P{GD12206}v27963	FBst0457219	VDRC	
UAS- *Tsr1* RNAi	y[1] v[1]; P{y[+t7.7] v[+t1.8]=TRiP.HMJ23662}attP40	RRID:BDSC_62305	BDSC	
UAS- *nclb* RNAi	w[1118]; P{GD4073}v12577	FBst0450559	VDRC	
UAS- *sta* RNAi	P{KK108871}VIE-260B (v101495)	FBst0473368	VDRC	
UAS- *sta* RNAi	y[1] sc[*] v[1] sev[21]; P{y[+t7.7] v[+t1.8]=TRiP.HMC06497}attP40/CyO	RRID:BDSC_77365	BDSC	
UAS- *Nopp140* RNAi	w[1118] P{GD12231}v27995	FBst0457235	VDRC	
UAS- *Nopp140* RNAi	y[1] sc[*] v[1] sev[21]; P{y[+t7.7] v[+t1.8]=TRiP.HMS00564}attP2	RRID:BDSC_33694	BDSC	
UAS- *RpS21* RNAi	P{KK109086}VIE-260B (v101558)	FBst0473431	VDRC	
UAS- *RpLP0* RNAi	y[1] v[1]; P{y[+t7.7] v[+t1.8]=TRiP.JF01335}attP2/TM3, Ser[1]	RRID:BDSC_31370	BDSC	
UAS- *RpL17* RNAi	y[1] v[1]; P{y[+t7.7] v[+t1.8]=TRiP.HMS03519}attP40	RRID:BDSC_54048	VDRC	
UAS- *CG5033* RNAi	y[1] sc[*] v[1] sev[21]; P{y[+t7.7] v[+t1.8]=TRiP.HMS02623}attP40	RRID:BDSC_42930	BDSC	
UAS- *CG5033* RNAi	y[1] sc[*] v[1] sev[21]; P{y[+t7.7] v[+t1.8]=TRiP.HMS02840}attP40	RRID:BDSC_44118	BDSC	
UAS- *pit* RNAi	y[1] v[1]; P{y[+t7.7] v[+t1.8]=TRiP.HMC06604}attP40	RRID:BDSC_80368	BDSC	
			
**Antibodies**				
**Name**		**Identifier**		**Source**
Monoclonal rat anti-E-cadherin DCAD2		RRID:AB_528120		Developmental Studies Hybridoma Bank
Monoclonal mouse anti-Singed Sn7C		RRID:AB_528239		Developmental Studies Hybridoma Bank
IgG1 Cross-Adsorbed Goat anti-Mouse, Alexa Fluor™ 568		Cat# A21124		Thermo Fisher Scientific
IgG (H+L) Cross-Adsorbed Goat anti-Rat, Alexa Fluor™ 488		Cat# A11006		Thermo Fisher Scientific
